# Synergistic Effect of Combination Interventions for Methicillin-Resistant *Staphylococcus aureus* Transmission Control in Nursing Homes: A Computation Modelling Evaluation with Heterogeneous Contact Mixing

**DOI:** 10.3390/antibiotics10030227

**Published:** 2021-02-24

**Authors:** Arthur Tang, Kin On Kwok, Vivian Wan In Wei, Hong Chen, Samuel Yeung Shan Wong, Wilson Wai Sun Tam

**Affiliations:** 1 Department of Software, Sungkyunkwan University, Suwon 16419, Korea; atang@skku.edu; 2 JC School of Public Health and Primary Care, The Chinese University of Hong Kong, Hong Kong 999077, China; vivian1628@cuhk.edu.hk (V.W.I.W.); yeungshanwong@cuhk.edu.hk (S.Y.S.W.); 3 Stanley Ho Centre for Emerging Infectious Diseases, The Chinese University of Hong Kong, Hong Kong 999077, China; 4 Shenzhen Research Institute, The Chinese University of Hong Kong, Shenzhen 518057, China; 5 Centre for Health Protection, Department of Health, Hong Kong 999077, China; ch459@ha.org.hk; 6 Alice Lee Centre for Nursing Studies, National University of Singapore, Singapore 117597, Singapore; nurtwsw@nus.edu.sg

**Keywords:** antimicrobial resistance, methicillin-resistant *Staphylococcus aureus* transmission, simulation and modelling, nursing home, interventions, synergistic effect

## Abstract

The endemic threat of methicillin-resistant *Staphylococcus aureus* (MRSA) in nursing homes poses a serious and escalating challenge to public health administration in infection control. Nursing homes are considered as major reservoirs for MRSA colonization, with considerable high levels of colonization prevalence. We employed a computation model to evaluate effects of three intervention scenarios on MRSA colonization prevalence rate in nursing homes. Simulations were conducted using a deterministic compartmental model featuring heterogeneous contact matrix between residents and health-care workers (HCWs). Contact parameters were derived from a nursing home survey. Three intervention scenarios were simulated: (1) hand-hygiene compliance by HCWs, (2) screening-and-isolation upon admission, and (3) implementing both interventions at the same time. For every 10% reduction in average contamination duration in HCWs, the estimated average reduction in prevalence rate was 1.29 percentage point compared with the prevalence rate before the intervention was implemented. Screening-and-isolation intervention resulted in an average reduction of 19.04 percentage point in prevalence rate (S.D. = 1.58; 95% CI = 18.90–19.18). In intervention scenario 3, synergistic effects were observed when implementing hand-hygiene compliance by HCWs and screening-and-isolation together. Our results provide evidence showing that implementing multiple interventions together has a synergistic effect on colonization prevalence reduction.

## 1. Introduction

Antimicrobial resistance (AMR) has become one of the major pressing public health threats and challenges globally. Antibiotics are widely used to combat life-threatening bacterial diseases. However, the imprudent use of antibiotics contributes to resistance among pathogenic microorganisms which in turn threatens the effective treatment of bacterial, parasitic, viral, and fungal infection. Methicillin-resistant *Staphylococcus aureus* (MRSA) is a multi-drug resistant strain of Staphylococcus aureus, and is regarded as one of the major AMR pathogens. Among the 18 drug-resistant threats identified by the United States Centers for Disease Control and Prevention, MRSA is regarded as a serious threat, where patients in healthcare settings frequently have severe or potentially life-threatening infections [1].

MRSA is commonly associated with the morbidity and mortality among elderly individuals. The overall incidence rates of MRSA infection among elderly individuals were consistently highest among other age groups globally [2,3,4]. Its strains were commonly identified in health care facilities, especially in nursing homes [5], which are considered as major reservoirs for MRSA [6]. MRSA was endemic at high levels in nursing homes [7,8]. Previous research works reported that MRSA colonization prevalence rate in nursing homes can be as high as 52% in the United States [9,10,11], significantly higher than that of 1.5% in the general population. Residents in nursing homes were shown to carry MRSA for a considerably long period of time: asymptomatic colonization could last for more than 3 years [12,13].

The endemic threat of MRSA in nursing homes along with the trend in global population aging pose a serious and escalating challenge to public health administration in infection control. Compared to acute care hospital settings, little was known about the epidemiology of MRSA in nursing homes. A primary effort in MRSA control of nursing homes is to drive down the endemic prevalence rate. Two key MRSA infection control measures for nursing-home are hand-hygiene by healthcare workers (HCWs) and screening followed by isolation for residents upon their admissions. One of the concerns for nursing homes’ administration is how to evaluate effectiveness among different interventions in reducing the colonization prevalence rate. As a natural experiment studying infectious diseases transmission is neither possible nor ethical, computational modelling becomes an essential tool to simulate how infectious diseases progress and to evaluate the impact of infection control policies with different intervention implementations.

The major transmission mode of MRSA infections is through direct contact with wounds, discharge, and soiled areas [14]. A recent systematic review on mathematical modelling of AMR summarizes computation models simulating MRSA transmission in nursing home settings [15]. Social contact in these models was mostly assumed to be homogeneous, except one modelling study in nursing home setting that explicitly represents the contact between residents and healthcare workers (HCWs) [6]. Our study further extended this computation model to evaluate the effect of two interventions to MRSA colonization prevalence: (1) hand-hygiene compliance by HCWs and (2) screening-and-isolation upon admission. Our study further evaluated a potential synergistic effect for implementing both of these two interventions together. We used the aggregate social contact information among residents and staff obtained in the previous contact survey [16] and demographics of nursing homes as parameters of our simulation model.

## 2. Results

**Figure 1:**
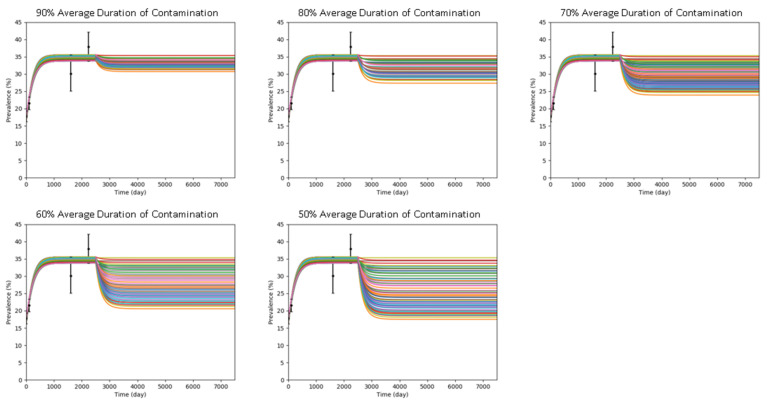
Plots of effect of five levels of hand-hygiene compliance to MRSA colonization prevalence. Hand-hygiene compliance interventions were implemented on Day 2501.

The results of the simulations in intervention scenario 1, hand-hygiene compliance for HCWs, are summarized in Table 1 and illustrated in Figure 1. It is further estimated that for every 10% reduction in average contamination duration, there is an average reduction of 1.29 percentage point in prevalence rate compared with the prevalence rate before the intervention was implemented.

**Figure 2:**
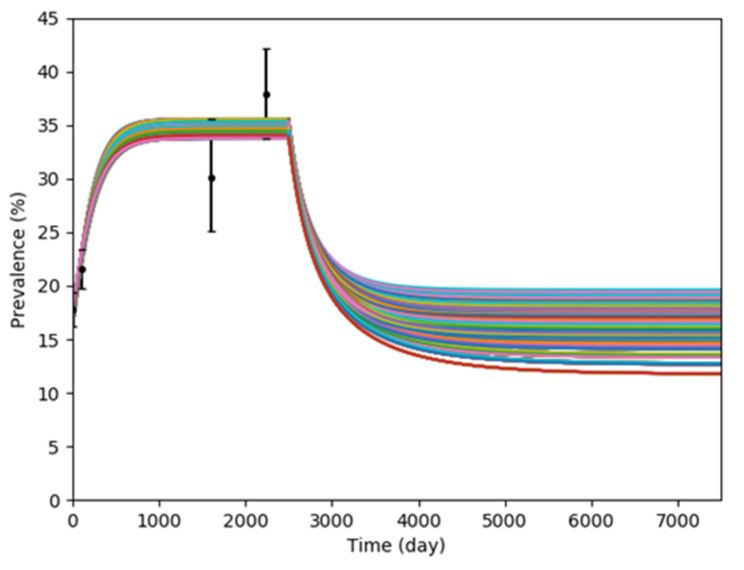
Plot of effect of screening-and-isolation upon admission to MRSA colonization prevalence.

The results of the simulations in intervention scenario 2, screening-and-isolation upon admission, are illustrated in Figure 2. Our simulation results estimated that the screening-and-isolation intervention results in an average reduction of 19.04 percentage point in prevalence rate (S.D. = 1.58; 95% CI = 18.90–19.18) compared with the prevalence rate before the intervention was implemented.

**Figure 3:**
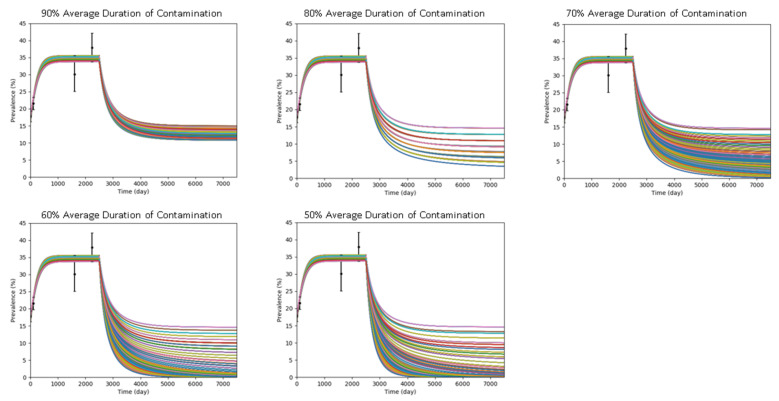
Plots of combined effect of hand-hygiene compliance and screening-and-isolation upon admission to MRSA colonization prevalence. Interventions were implemented on Day 2500.

The results of the simulations in intervention scenario 3, implementing both interventions at the same time, are illustrated in Figure 3 and summarized in Table 2. A synergistic effect is observed; the effect of scenario 3 is larger than the combined effect of scenario 1 and scenario 2.

## 3. Discussion

Close contact is the major transmission route of MRSA infections. The primary considerations in this study are how to represent the heterogeneity of contact mixing between residents and HCWs in the model, and the availability of social contact matrix data for parameters estimation. The modeling framework of this study employed explicit representation of the heterogeneous contact mixing between residents and HCWs. The contact parameters of this study were estimated based on the results of a nursing home survey conducted for residents and HCWs of 53 nursing homes. These approaches provide more legitimate simulation results of MRSA transmission in nursing homes. The model and methodology of this study also provide an instantaneous framework to evaluate hypothetical scenarios and to conduct “what-if” analyses. Nursing home administrators can employ the methodology of our study to make informed judgment and decision on infection control strategies accordingly.

Our simulation results suggest that the two simulated interventions, (1) hand-hygiene compliance for HCWs and (2) screening-and-isolation upon admission, are effective in driving down MRSA colonization prevalence. Quantitative data shows that every 10% reduction in average contamination duration for HCWs results in a reduction of 1.29 percentage points in prevalence on average. The screening-and-isolation upon admission intervention was also shown to be effective in driving prevalence down, with a 19.04 percentage point reduction on average. In intervention scenario 3, a synergistic effect is observed when implementing both interventions together. The magnitude of effect in prevalence reduction in scenario 3 is larger than the sum of the magnitude of effect in scenarios 1 and 2. The synergistic effects observed by implementing both interventions together in scenario 3 is quite encouraging. We are not aware of any evidence showing this kind of synergistic effect in prior literature. This result should be brought to broader attention as hand-hygiene based intervention is relatively convenient to be implemented and is shown to be effective in bringing down MRSA colonization prevalence. Our simulation results provide quantitative data for nursing home administrators in interventions planning, design, adjustment, and effect estimation and comparison.

To the best of our knowledge, the latest systematic review about MRSA transmission control in nursing homes was conducted in 2013 [17]. One clinical study [18] was included in this systematic review. We further identified two additional clinical studies about MRSA transmission control in nursing homes [19,20]. Currently, there is a lack of prior clinical studies evaluating effectiveness of different interventions on MRSA prevalence in nursing home. While there exists a sizable amount of clinical studies evaluating MRSA intervention effectiveness in hospital settings, MRSA epidemiology in hospitals is quite different from that in nursing homes. Infection control measures for MRSA used in hospital settings should be applied with caution to nursing home before being validated by clinical data.

The three prior clinical studies evaluating MRSA transmission control in nursing homes employed different intervention strategies, including hand-hygiene training program for HCWs, screening-and-isolation, and environmental cleaning. The study by Baldwin et al. [18] employed hand-hygiene training program for HCWs, screening-and-isolation, and environmental cleaning in their intervention program, and their interventions program has no significant effect on MRSA prevalence. The study by Hequet et al. [19] employed hand-hygiene and training program for HCWs in their intervention program, and their intervention program has no significant effect on MRSA prevalence. The study by Peterson et al. employed hand-hygiene training program for HCWs, screening-and-isolation, and environmental cleaning in their intervention program, and their intervention program has a significant reduction on MRSA prevalence with the interventions employed. Results in intervention effectiveness among these three clinical studies are inconclusive. Prior research in MRSA control in hospital settings suggests that hand hygiene compliance is crucial in reducing healthcare associated infections [21]. The results from our simulation also demonstrated that different level of hand hygiene compliance results in different levels of long term MRSA prevalence reduction. Intervention compliance could be the crucial factor in MRSA control, rather than the choice of intervention methods [22].

Hand-hygiene intervention in health-care settings is beyond merely providing hand-hygiene facilities and implementing policy for HCWs. A prior study [23] reported that a one-hour lecture plus 30-min hands-on session could effectively improve the hand-hygiene compliance from 9.3% to 30.4%. The World Health Organization (WHO) published guidelines in 2009 for implementing and evaluating hand hygiene programmes in healthcare settings [24]. The guidelines had identified five components to be implemented in healthcare-setting, including (1) using soap and water or alcohol-based hand rub at point of care or carried by the HCWs, (2) training and education, (3) observation and performance feedback, (4) reminders, and (5) administrative support/institutional safety climate. A recent systematic review reported that multimodal interventions that include some or all strategies recommended in the WHO guidelines might slightly improve hand hygiene compliance [25]. As to screening-and-isolation, although it was advocated by many researchers [26,27], studies in nursing homes were limited, but some studies reported the effectiveness of such measure in hospital settings [28,29].

## 4. Materials and Methods

### 4.1. The Computational Model

**Figure 4:**
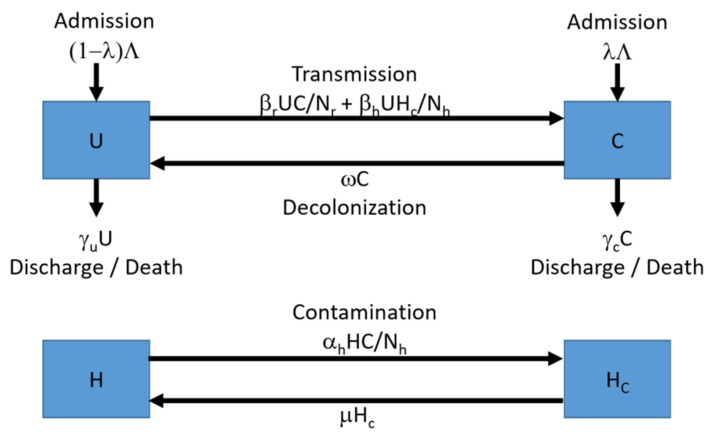
Schematic of the transmission model.

A two-level compartmental model with deterministic framework was employed. Nursing home residents are considered as hosts of MRSA, and HCWs are considered as transmission vectors. Residents are categorized into two mutually exclusive states: (a) uncolonized or (b) colonized; whereas HCWs are categorized into two mutually exclusive states: (c) contaminated or (d) uncontaminated. The model features the residents’ admission from community/hospital and discharge/death of the residents who are susceptible and colonized. Different routes of transmission were taken into account via the close contact among HCWs and residents: (i) HCWs-residents contacts, (ii) residents-residents contacts, and (iii) residents-HCWs contacts. Two types of transmission were defined in the model: Colonization and Contamination. MRSA can be transmitted to uncolonized residents by contact made with colonized residents or contaminated HCWs. Uncontaminated HCWs can be contaminated by contact made with colonized residents. Schematic of the transmission model is illustrated in Figure 4.

The model is described with the following set of nonlinear differential equations:

(1) $$\frac{dU\left(t\right)}{dt}=\;-\frac{\beta_{r-r}U\left(t\right)\;C\left(t\right)}{N_{r}}-\frac{\beta_{h-r}U\left(t\right)H_{c}\left(t\right)}{N_{h}}+\omega C\left(t\right)+\left(1-\lambda \right)\Lambda -\gamma_{u}U\left(t\right)$$

(2) $$\frac{dC\left(t\right)}{dt}=\;\frac{\beta_{r-r}U\left(t\right)\;C\left(t\right)}{N_{r}}+\frac{\beta_{h-r}U\left(t\right)\;H_{c}\left(t\right)}{N_{h}}-\omega C\left(t\right)+\lambda \Lambda -\gamma_{c}C\left(t\right)$$

(3) $$\frac{dH\left(t\right)}{dt}=\;-\frac{\beta_{r-h}H\left(t\right)\;C\left(t\right)}{N_{r}}-\frac{\beta_{h-h}H\left(t\right)\;H_{c}\left(t\right)}{N_{h}}+µH_{c}\left(t\right)$$

(4) $$\frac{dH_{c}\left(t\right)}{dt}=\frac{\beta_{r-h}H\left(t\right)\;C\left(t\right)}{N_{r}}-µH_{c}\left(t\right)$$

where *U*(*t*), *C*(*t*), *H*(*t*), and *H_c_*(*t*) denote the population size of uncolonized-residents, colonized-residents, uncontaminated-HCWs, and contaminated-HCWs, respectively in a nursing home at time t; 1/μ denotes the mean duration of contamination, while 1/ω denotes the mean duration of colonization. N_r_ denotes the population of residents and N_h_ denotes the population of healthcare-workers, where N_r_ = U(t) + C(t) and N_h_ = H(t) + H_c_(t).

The average length of stay of colonized residents and uncolonized residents are denoted by γ_u_ and γ_c_, respectively. Residents are admitted at the rate of Λ, and the probability of admitted residents being colonized is λ. It is assumed that the number of residents and HCWs remain constant, and the occupancy rate is 100%. Therefore, residential admission rate equals to residents’ discharge rate; i.e., Λ = γ_u_U + γ_c_C.

Three transmission rates were defined in the model. β_r-r_ and β_h-r_ denoted the residents-to-residents transmission rate and HCWs-to-residents transmission rate respectively, whereas β_r-h_ denoted the residents-to-HCWs transmission rate. The values of these three transmission rates were defined as the estimated number of contacts multiplied by the transmission probability via contact, as summarized in Table 3 and Table 4. Three types of contact and transmission probability were defined; a_r-r_, a_r-h_, and a_h-r_ denote the average number of residents-to-residents contact, residents-to-HCWs contact, and HCWs-to-residents contact, respectively; p_r-r_, p_r-h_, and p_h-r_ denote the transmission probability via residents-to-residents contact, residents-to-HCWs contact, and HCWs-to-residents contact, respectively.

### 4.2. Parameterization

#### 4.2.1. Parameter Identification

Fourteen parameters were incorporated in the model. Values of 12 of the 14 parameters were identified in the parameterization process. Values of 2 parameters cannot be identified and were estimated. Table 5 summarizes the 14 parameters.

#### 4.2.2. Parameter Estimation

No best estimates of two key parameters were identified from prior studies: (a) transmission probability via residents-to-residents contact and (b) transmission probability via HCWs-to-residents contact. We estimated these two parameters by fitting the mathematical transmission model to the prevalence data. Four MRSA point-prevalence rates and their 95% confidence interval (CI) of nursing homes in Hong Kong were identified from prior studies (Table 6) [30,34,35,36]. We denoted the date of these four point-prevalence rates as Day 1, Day 108, Day 1600, and Day 2239.

**Figure 5:**
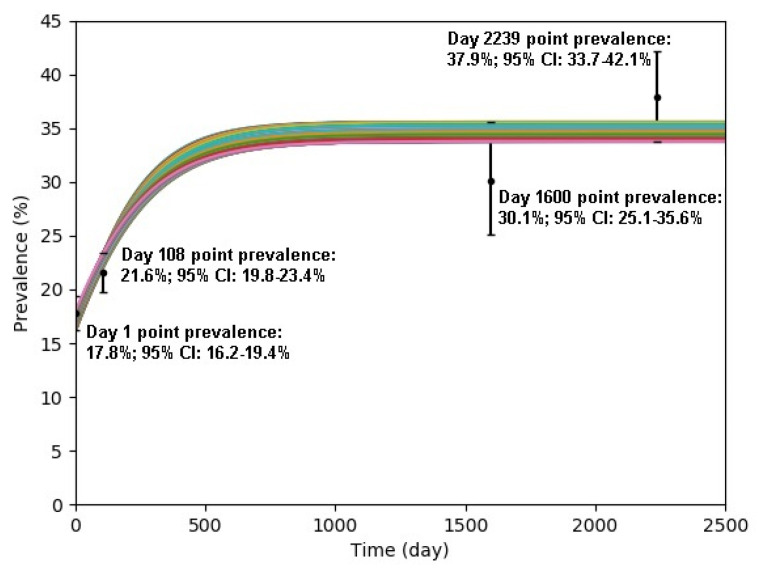
Plot of 487 simulation results that fitted within 95% CI of the four point-prevalence rates on their respective day.

Simulations using the computational model described in the previous section were conducted by varying the two unknown transmission probabilities and the prevalence of Day 1. The combinations of the three variables used in the simulations are constructed based on 3-dimensional hypercube, varying the two transmission probabilities from 0% to 100% (with 0.1% increments) and the prevalence of Day 1 from 16.2% to 19.4% (with 0.1% increment); 33,066,033 simulations (1001 × 1001 × 33) were conducted. Each simulation was conducted to simulate prevalence for 2500 days where the prevalence rates reached equilibrium. Of the 33,066,033 simulation generated results, 487 fitted within the 95% confidence interval of the four point-prevalence rate on their respective day. These 487 sets of parameters were used to further conduct simulations of interventions. Figure 5 illustrates simulation results of these 487 simulations.

### 4.3. Simulations of Interventions

Three types of intervention scenarios were evaluated using the computational model: (1) hand-hygiene compliance by HCWs, (2) screening-and-isolation upon all admission, and (3) implementing both interventions at the same time. Assuming interventions implemented on Day 2501, we simulated the long term impact of different hypothetical intervention scenarios on MRSA transmission dynamics. The 487 sets of parameters that fitted within 95% CI of the four point-prevalence rates were used as the input parameters for the computational model. Effects of interventions were evaluated by comparing the point prevalence of Day 2500 and Day 7500 for each of the simulations.

#### 4.3.1. Intervention Scenario 1: Hand-Hygiene Compliance by HCWs

Hand-hygiene compliance by HCWs was simulated by reducing the average contamination duration 1/μ. Five levels of hand-hygiene compliance by HCWs were evaluated by reducing the average contamination duration by 10%, 20%, 30%, 40%, or 50%. Computational model defined in the previous section was used to simulate effects of hand-hygiene by HCWs compliance to prevalence in our study; 2435 simulations (5 levels of compliance ×487 set of parameters) were conducted for this scenario.

#### 4.3.2. Intervention Scenario 2: Screening-and-Isolation Upon Admission

**Figure 6:**
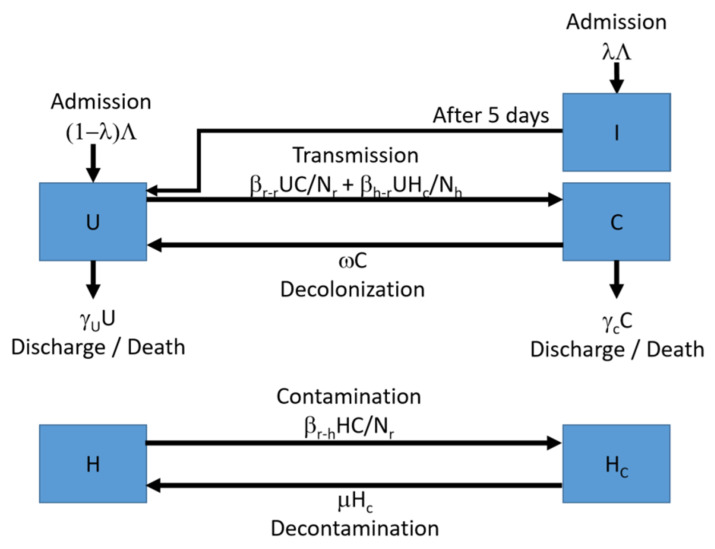
Schematic of the extended model for screening-and-isolation upon admission.

The model was further extended in order to study effects of screening-and-isolation upon admission to prevalence rate. We assumed a 100% screening success rate, a 5-day isolation period, and a 100% decolonization rate after 5 days in isolation. An additional compartment was added in the model in order to represent the screening-and-isolation upon admission. All colonized admissions were put into an isolation compartment I, and they were forwarded to compartment U after 5 days. Schematic of the extended model is illustrated in Figure 6; 487 simulations were conducted for this scenario.

#### 4.3.3. Intervention Scenario 3: Implementing Both Interventions at the Same Time

Simulation of intervention scenario 2 was run with five levels of hand-hygiene compliance by HCWs (reducing the average contamination duration by 10%, 20%, 30%, 40%, or 50%); 2435 simulations were conducted for this scenario.

## 5. Conclusions

We presented a computational model with heterogeneous social contact mixing to simulate the transmission of MRSA in nursing homes. This approach can be used to conduct scenario analyses for MRSA transmission in nursing homes, and can also be generalized to other settings including hospitals and households as well as multi-drug resistance organisms such as extended spectrum beta-lactamases and Carbapenem-resistant enterobacteriaceae. Several limitations of the study are noteworthy. First, the potential super-spreading events and transmissibility variation in individual or sub-groups were not considered in this model. Second, this study mainly employed the survey data of nursing homes in Hong Kong to parameterize the model. Region or country specific data including point prevalence, characteristics of nursing home, and social mixing matrix is needed to study the situations in different countries/regions. Finally, we are not aware of any prior study about the efficacy of MRSA screening and decolonization of nursing homes and 100% efficacy was assumed in this study. Further study is needed to parameterize these two factors accurately.

## Figures and Tables

**Table 1:** Effect of five levels of hand-hygiene compliance to Methicillin-Resistant *Staphylococcus aureus* (MRSA) colonization prevalence.

Hand-Hygiene Compliance Level	Average Reduction in Prevalence (In Percentage Point)	Standard Deviation	95% C.I.
10% reduction in average contamination duration for HCWs	1.38	0.92	1.30–1.46
20% reduction in average contamination duration for HCWs	2.84	1.91	2.67–3.01
30% reduction in average contamination duration for HCWs	4.37	2.93	4.10–4.62
40% reduction in average contamination duration for HCWs	5.91	3.96	5.56–6.26
50% reduction in average contamination duration for HCWs	7.44	4.93	7.01–7.88

**Table 2:** Combined effect of hand-hygiene compliance and screening-and-isolation upon admission to MRSA colonization prevalence.

Hand-Hygiene Compliance Level	Average Reduction in Prevalence (In Percentage Point)	Standard Deviation	95% C.I.
10% reduction in average contamination duration for HCWs	21.99	0.65	21.93–22.05
20% reduction in average contamination duration for HCWs	25.34	2.60	25.11–25.57
30% reduction in average contamination duration for HCWs	28.31	4.14	27.94–28.67
40% reduction in average contamination duration for HCWs	30.00	4.49	29.60–30.40
50% reduction in average contamination duration for HCWs	30.94	4.44	30.55–31.34

**Table 3:** The three transmission rates defined in the computational model.

Description	Symbol	Value
Residents-to-residents transmission rate	β_r-r_	a_r-r_p_r-r_
HCWs-to-residents transmission rate	β_h-r_	a_h-r_p_h-r_
Residents-to-HCWs transmission rate	β_r-h_	a_r-h_p_r-h_

**Table 4:** The six parameters used in the three transmission rates.

Description	Symbol
Average number of residents-to-residents contact	a_r-r_
Average number of HCWs-to-residents contact	a_h-r_
Average number of residents-to-HCWs contact	a_r-h_
Transmission probability via residents-to-residents contact	p_r-r_
Transmission probability via HCWs-to-residents contact	p_h-r_
Transmission probability via residents-to-HCWs contact	p_r-h_

**Table 5:** List of the 14 parameter values used in the computational model.

Description	Symbol	Value	Reference
Number of residents	N_r_	75.10	[16]
Number of HCWs	N_hcw_	8.61	[16]
Probability of admission of colonized residents	Λ	15.8%	[30]
Average duration of colonization (days)	1/ω	268.8	[31]
Average length of stay for uncolonized residents (days)	1/γ_u_	233.89 *	[30,32]
Average length of stay for colonized residents (days)	1/γ_c_	233.89 *	[30,32]
Average contamination duration (hours)	1/μ	4.1 *	[16]
Average number of residents-to-residents contact	a_r-r_	0.2	[16]
Average number of HCWs-to-residents contact	a_h-r_	1.2	[16]
Average number of residents-to-HCWs contact	a_r-h_	12.7	[16]
Average number of HCWs-to-HCWs contact	a_h-h_	0.6	[16]
Transmission probability via residents-to-residents contact	p_r-r_	Not identified	
Transmission probability via HCWs-to-residents contact	p_h-r_	Not identified	
Transmission probability via residents-to-HCWs contact	p_r-h_	20%	[33]

* Average length of stay for uncolonized residents and average length of stay for colonized residents were calculated based on data presented in Kwok et al. [18]. Average contamination duration was calculated based on data presented in Kwok et al. [15]. Detail of calculations of these three parameters is included in Supplementary Material S1.

**Table 6:** Point-prevalence of MRSA in Hong Kong nursing homes.

Study Period	Mid-Date of Study Period	Day Count	Prevalence	95% C.I.	Reference
3 March–26 September 2011	14 June 2011	1	17.8%	16.2–19.4%	[34]
1 July–31 December 2011	30 September 2011	108	21.6%	19.8–23.4%	[30]
1 September–31 December 2015	31 October 2015	1600	30.1%	25.1–35.6%	[35]
1 July–31 August 2017	31 July 2015	2239	37.9%	33.7–42.1%	[36]

## Data Availability

Data sharing not applicable. No new data were created or analyzed in this study. Data sharing is not applicable to this article.

## Supplementary Materials

The following are available online at https://www.mdpi.com/2079-6382/10/3/227/s1, Supplementary Material S1: Parameterization.
