# Supplementary material for: Synergistic Effect of Combination Interventions for Methicillin-Resistant Staphylococcus aureus Transmission Control in Nursing Homes: A Computation Modelling Evaluation with Heterogeneous Contact Mixing
Source: Antibiotics (Basel). 2021 Feb 24;10(3):227. doi: 10.3390/antibiotics10030227 (PMC7996172; doi:10.3390/antibiotics10030227)
Supplement: Supplementary file 1 [file antibiotics-10-00227-s001.pdf]

antibiotics

MDPI

Supplementary Material S1. Parameterization

Three parameters were not directly available from prior studies, and were calculated based on data presented in prior studies. These three parameters are: (1) Average length of stay for uncolonized residents, (2) average length of stay for colonized residents, and (3) average contamination duration for health-care workers (HCWs).

Average length of stay for uncolonized residents and average length of stay for colonized residents

According to a prior study by Kwok et al. (2017), for people older than 65 years who stayed in a hospital:

Their average day of stay in a hospital is 5.92 days.

They were discharged from hospital 2.54 times on average.

The average days outside of hospital for people older than 65 years who stayed in a hospital is calculated as:

365 – (5.92 days × 2.54 times) = 349.96 days

The average duration outside of hospital for people older than 65 years who stayed in a hospital is calculated as:

349.96 days / 2.54 times = 137.78 days

According to a prior study by Cheng et al. (2013), 42.3% of long-term care facility residents in the study had no hospitalization history in past 12 months; 57.7% of long-term care facility residents stayed in a hospital at least once.

We assume that residents of long-term care facilities are primarily older than 65 years. Therefore, the average length of stay in long-term care facilities is calculated as:

57.7% x 137.78 days + 42.3% x 365 days = 233.89 days

Average contamination duration for HCWs

According to a prior study by Kwok et al. (in preparation), the average HCW working hours per day is 8.2 hours. We assume the average contamination duration as half of the daily working hours, i.e., 4.1 hours.

Reference

1. Cheng V.C.; Tai J.W., Wong Z.S.; Chen J.H.; Pan K.B.; Hai Y., et al. Transmission of methicillin-resistant Staphylococcus aureus in the long term care facilities in Hong Kong. *BMC Infect Dis* **2013,** *13*, 205.
2. Kwok C.L.; Lee, C.K.M.; Lo, W.T.L.; Yip, P.S.F. The Contribution of Ageing to Hospitalisation Days in Hong Kong: A Decomposition Analysis. *Int J Health Policy Manag* **2017**, *6*, 155–164.
3. Kwok K.O.; Chan H.; Wei V.W.I.; Tang A.; Riley S. A longitudinal contact survey in residential care homes for the elderly in Hong Kong. (In preparation).
